# The complete mitochondrial genome analysis of *Haemaphysalis hystricis* Supino, 1897 (Ixodida: Ixodidae) and its phylogenetic implications

**DOI:** 10.1515/biol-2022-0875

**Published:** 2025-03-18

**Authors:** Zhong-Bo Li, Min Xiang, Tian Yang, Hui Hu, Ming Shu, Cui-qin Huang

**Affiliations:** College of Animal Science and Technology, HuaiHua Vocational and Technical College, Huaihua, Hunan, 418000, PR China; College of Life Science, Longyan University, Longyan, Fujian, 364012, PR China; Engineering Research Center for the Prevention and Control of Animal Original Zoonosis, Fujian Province University, College of Life Science, Longyan University, Longyan, Fujian, 364012, PR China

**Keywords:** *Haemaphysalis hystricis*, mitochondrial genome, sequence characteristics, codons usage, phylogenetic analysis

## Abstract

In order to study the sequence characteristics, gene order, and codon usage of the mitochondrial genome of *Haemaphysalis hystricis*, and to explore its phylogenetic relationship, a total of 36 *H. hystricis* isolated from dogs were used as sample in this study. The mitochondrial genome of a *H. hystricis* was amplified with several pairs of specific primers by PCR, and was sequenced by first generation sequencing. The mitochondrial genome of *H. hystricis* was 14,719 bp in size, and it contained 37 genes including 13 protein coding genes (PCGs), 22 transfer RNA genes (tRNAs), 2 ribosomal RNA genes (rRNAs), and AT-rich region. Each PCG sequence had different lengths, the sequence longest and shortest gene were *nad*5 (1,652 bp) and *atp*8 (155 bp), respectively, among the 13 PCGs. All PCGs used ATN as their initiation codon, 10 of 13 PCGs used TAN as their termination codon, and 3 of which had incomplete termination codon (TA/T). Most of the 22 tRNAs with different sizes could form the classical cloverleaf structures expect for *tRNA*-*Ala*, *tRNA*-*Ser1*, *tRNA*-*Ser2*, and *tRNA*-*Glu*, and there were base mismatch (U-U and U-G) in all the 22 tRNAs sequences. Two rRNAs, namely *rrnL* and *rrnS*, had different lengths, *rrnL* located between *tRNA*-*Leu1* and *tRNA*-*Val*, and *rrnS* located between *tRNA*-*Val* and *tRNA*-*Ile*, respectively. Two AT (D-loop) control areas with different lengths were in the mitochondrial genome, the NCRL was located between *tRNA*-*Leu2* and *tRNA*-*Cys*, and the NCRS was located between *rrnS* and *tRNA*-*Ile*. The complete mitochondrial genome sequence of *H. hystricis* was AT preferences, and the gene order is the same as that of other *Haemaphysalis* family ticks. However, phylogenetic analysis showed that *H. hystricis* was most closely related to *Haemaphysalis longicornis* among the selected ticks. The mitochondrial genome not only enriches the genome database, provides more novel genetic markers for identifying tick species, and studying its molecular epidemiology, population genetics, systematics, but also have implications for the diagnosis, prevention, and control of ticks and tick-borne diseases in animals and humans.

## 1 Introduction

Ticks, as an obligatory blood-feeding parasites [1,2], could parasitize humans and various animals including birds, mammalians, and crawls [3,4], and can also transmit numerous pathogenic microorganisms such as bacteria, fungi, viruses, and protozoa [5,6], which can lead to disease in invaded animals [7,8], and sometimes even cause death [9]. At present, ticks are classified into three families, namely the Argasidae, the Ixodidae, and the Nuttalliellidae, which encompass 867 species of tick that are recorded in the world [10]. The Ixodidae family, comprising 682 species, is the largest among the three families, which encompass 5 subfamilies and 13 genus [11]. *Haemaphysalis hystricis* (*H. hystricis*) belongs to Acarina, Ixodidae, and Haemaphysalis [12], which usually parasitizes on dogs and other hosts including humans at times [13,14]. As a common and important vector, *H. hystricis* carries and transmits a number of pathogens, including *Babesia canis*, *Ehrlichia canis*, and *Rickettsia conorii* [15,16], posing a huge threat to the health of both humans and animals [17]. Moreover, *H. hystricis* is widely distributed in China and others countries [12], and has become a dominant tick species in some areas of China. However, current knowledge about *H. hystricis* is only limited to its morphology and biology, and limited molecular information of this tick is reported, especially in genetics and molecular epidemiology owing to the lack of genome information and suitable genetic markers. Thus, the study on mitochondrial genome of *H. hystricis* is a key step for finding the genetic marker applying to its molecular epidemiology, population genetics, and systematics study, and have implications for studying the diagnosis, prevention, and control of ticks and tick-borne diseases in animals and humans in future [11,18].

Generally, for identification and classification of ticks, the traditional taxonomic method according to morphological structure of parasites is still a main method [19,20]. It can clearly display the morphological characteristics of various parts of the insect body but not study the insects’ gene variation, genetic structure, and phylogenetic relationship at the molecular level and the traditional taxonomic method is highly dependent on the integrity of the sample and the experience of the appraiser. However, when insects are at different developmental stages or when they have extremely similar morphological structure, the traditional taxonomic method cannot effectively identify the parasite species [21]. Therefore, it is a crucial step in parasitology research to apply others methods to accurately identify the parasite species. With the rapid development of molecular biotechnology, especially the popularization of PCR technology, the parasitology study is also developing toward the molecular level. Currently, as more and more genome information of parasite is deposited to GenBank, including mt genome, ribosomal genome, and RNAs, various gene markers used in parasite species identification are found, including *cox1*, *nad1*, *nad4*, *18S rRNA*, *ITS*-1, and *ITS*-2 [22,23]. However, some researchers believe that the complete mt genome sequence supplied information is more than that of fragment gene sequence [24], hence the mt genome sequence is more suitable for analyzing parasitic gene variation, genetic structure, and phylogenetic study [25–27]. Mt genome of metazoan (expect for lice and flea) is a double stranded circular DNA molecule [28–30], which has rapid evolutionary rate, matrilineal inheritance, multiple copies, and no genetic recombination characteristics [28,31]. Currently, mt genome data have become a valuable source for studying parasitology, which provide numerous molecular genetic markers. These genetic markers from mt genome sequence have been widely applied for the genetics and molecular identification of many zoonotic ectoparasites including ticks and t insects that suck animal blood [32–34]. Therefore, based on the study status of mt genome of *H. hystricis*, we sequenced the complete mt genome of a tick representative *H. hystricis* collected from a hound dog in Huaihua district, Hunan province, China, and analyzed its sequence characteristics, gene order, and codons usage. The phylogenetic relationship of *H. hystricis* and other ticks from Haemaphysalis family was performed by the concatenated amino acid sequence of 13 protein-coding genes (PCGs) in this study. Thereby, the aims of this study were (i) to determine the complete mt genome of *H. hystricis*; (ii) to analyze its sequence characteristic, gene order, codon usage, and the secondary structure of tRNAs and rRNAs; (iii) to reveal the phylogenetic relationship of *H. hystricis* and other ticks; and (iv) to provide novel mitochondrial resources and genetic markers to this ectoparasite.

## 2 Materials and methods

### 2.1 Parasites and DNA extraction

A total of 36 adult ticks of *H. hystricis* were obtained from a hound dog in Huaihua district, Hunan province, China. After being removed from the surface of the dog’s body, ticks were washed three times with physiological saline in a glass culture dish, and were identified morphologically for species under an optical microscope according to the existing keys and descriptions [12]. The genomic DNA was extracted from an individual sample using the blood and tissue DNA extraction kit (TIANGEN) and sodium SDS/proteinase K according to the manufacturer’s recommendations, was then purified by the DNA purification kit (TIANGEN), and was finally eluted into 50 µL using ddH_2_O. The rest of the ticks were fixed in 70% (v/v) ethanol at room temperature, and the extracted DNA was stored at −20°C until use.


**Ethical approval:** The research related to animal use has been complied with all the relevant national regulations and institutional policies for the care and use of animals and has been approved by the Animal Ethics Committee of HuaiHua Vocational and Technical College.

### 2.2 PCR amplification and sequencing

The complete mt genome of *H. hystricis* was divided into five long fragments to amplify by long PCR with ten pairs of primers as shown in Table 1. The primers to amplify target genes were designed based on that of conserved gene sequences on mt genome of other ticks that have a closer phylogenetic relationship, and were then biosynthesized by BGI-Shenzhen (Shenzhen, China). The long gene fragments of mt genome of *H. hystricis* were amplified with specific primers by long PCR. PCR amplifications were performed in a volume of 50 μL containing 2 mM of MgCl_2_, 0.2 mM each of deoxyribonucleoside triphosphate, 2.5 μL of 10× rTaq buffer, 2.5 μM of each primer, 1.25 U rTaq polymerase (Takara), 1 μL of DNA sample, and 22 μL of ddH_2_O. The cycling conditions consisted of predenaturation at 94°C for 5 min, followed by 35 cycles of denaturation at 94°C for 1 min, annealing at 53–56°C for 45 s and extension at 72°C for 1.5 min, followed by a final extension at 72°C for 5 min. After amplification, the PCR products (5 μL) were examined on a 1.5% agarose gel and visualized under a UV transilluminator. The PCR products were bidirectionally sequenced with a primer walking strategy by Shenggong Biotechnology (Shanghai, China).

**Table 1:** Primers used to amplify the complete mt genome of *H. hystricis*

Amplified region	Sequence (5′–3′)
*cox*1	F: TTTAGTTGAAAGAGGAGCCG
	R: TGATTCCTGTTAGTCCTCCAAC
*nad*1	F: TTCTTCAATAGCTTAAATAATTCC
	R: GTTTTATTAANAGNAGCTTTTTTCACTC
*rrnS*	F: GCACTTTCCAGTACTTTAACTTTG
	R: TCTCTAGTTAATTTNGTGCCAGC
*cyt*b	F: GTTATTCCAACTTTTAAATTCAATG
	R: TTGGTGTAATAATAAAAAATAAGAAG
*cox*1-*nad*1	F: TGATTCTTTGGACACCCAGA
	R: CATTCAAAGTATTCTCTTATTGGATC
*nad*1**-** *rrnS*	F: CCTGCCTTATTTGGTCCTTT
	R: GGCGGTATTTTAAGCTTTTC
*rrnS*-*cytb*	F: GCACTTTCCAGTACTTTAACTTTG
	R: TTGGTGTAATAATAAAAAATAAGAAG
*cyt*b-*cox*1	F: GTTATTCCAACTTTTAAATTCAATG
	R: TGATTCCTGTTAGTCCTCCAAC

### 2.3 Sequence analyses

Each obtained sequence was corrected by Chromas software. Sequences were assembled manually and aligned by comparing with the whole mt genome sequences of *Haemaphysalis longicornis* and *Haemaphysalis flava* available from the GenBank. The gene boundaries in this mt genome were identified by the computer program MAFFT 7.122. Based on the comparison with that of ticks reported previously, the translation initiation and translation termination codons were identified using the computer program Expasy (https://web.expasy.org/translate) [35]. For analyzing sequence characteristics, the four bases A, T, G, and C contents were calculated by the computer program DNAMAN, and the *A* + *T* and *G* + *C* contents were also calculated, respectively, based on *A*, *T, G*, and *C* contents. Most of the putative secondary structures of tRNAs were identified by the computer program tRNAscan-SE [36], and those of the tRNAs’ secondary structure unidentified by tRNAscan-SE was recognized by eye [28]. Meanwhile, all anticodon sequences of tRNA genes were recognized manually. However, two rRNA genes with different sizes in this mt genome were predicted by comparing with the mt genome sequences of *H. longicornis* and *H. flava* reported previously.

### 2.4 Phylogenetic analyses

Thirteen PCG sequences and two rRNAs gene sequences were concatenated into a single amino acid sequence according to the gene order in mt genome of *H. hystricis*. However, 18 formerly sequenced ticks’ mt genomes downloaded from the GenBank database (https://www.ncbi.nlm.nih.gov/genbank), including *H. longicornis* (MG721210), *Ixodes persulcatus* (NC004370), *Ixodes hexagonus* (AF081828), *Ixodes holocyclus* (AB075955), *Ixodes pavlovskyi* (NC023831), *Haemaphysalis formosensis* (NC020334), *Haemaphysalis concinna* (NC034785), *Haemaphysalis inermis* (NC020335), *Dermacentor nuttalli* (NC028528), *Dermacentor silvarum* (NC026552), *Dermacentor nitens* (NC023349), *Amblyomma triguttatum* (AB113317), *Amblyomma cajennense* (OP901707), *Rhipicephalus australis* (KC503255), *Rhipicephalus simus* (KJ739594), *Rhipicephalus sanguineus* (AF081829), *Rhipicephalus microplus* (KP143546), and *H. flava* (MG604958) were used as inner group. Later, the mt genome sequence of *Ornithodoros savignyi* (KJ133599) and *Argas persicus* (OM368320) downloaded from GenBank were also used as out-group. The computer software MAFFT 7.122 was used to align all amino acid sequences, and the Gblocks online server (http://molevol.cmima.csic.es/castresana/Gblocksserver.html) was used to align and exclude the ambiguous regions in the concatenated sequence using less stringent selection options under setting default parameters [37]. These concatenated sequences were subjected to phylogenetic analysis using maximum likelihood (ML) and Bayesian Inference (BI; MrBayes), respectively. The computer software ProtTest 2.4 was used to select the most suitable model of evolution, and the GTR + *G* + *R* model was selected based on the Akaike information criterion [38]. The computer software PhyML 3.0 was used to conduct the ML and BI analysis using the subtree pruning and regrafting method, and the ML and BI trees were constructed by FigTreev.1.4 with the default parameters [39]. In addition, Bootstrap frequency was calculated using 100 bootstrap replicates.

## 3 Results and analysis

### 3.1 Mitogenome organization and composition

**Figure 1:**
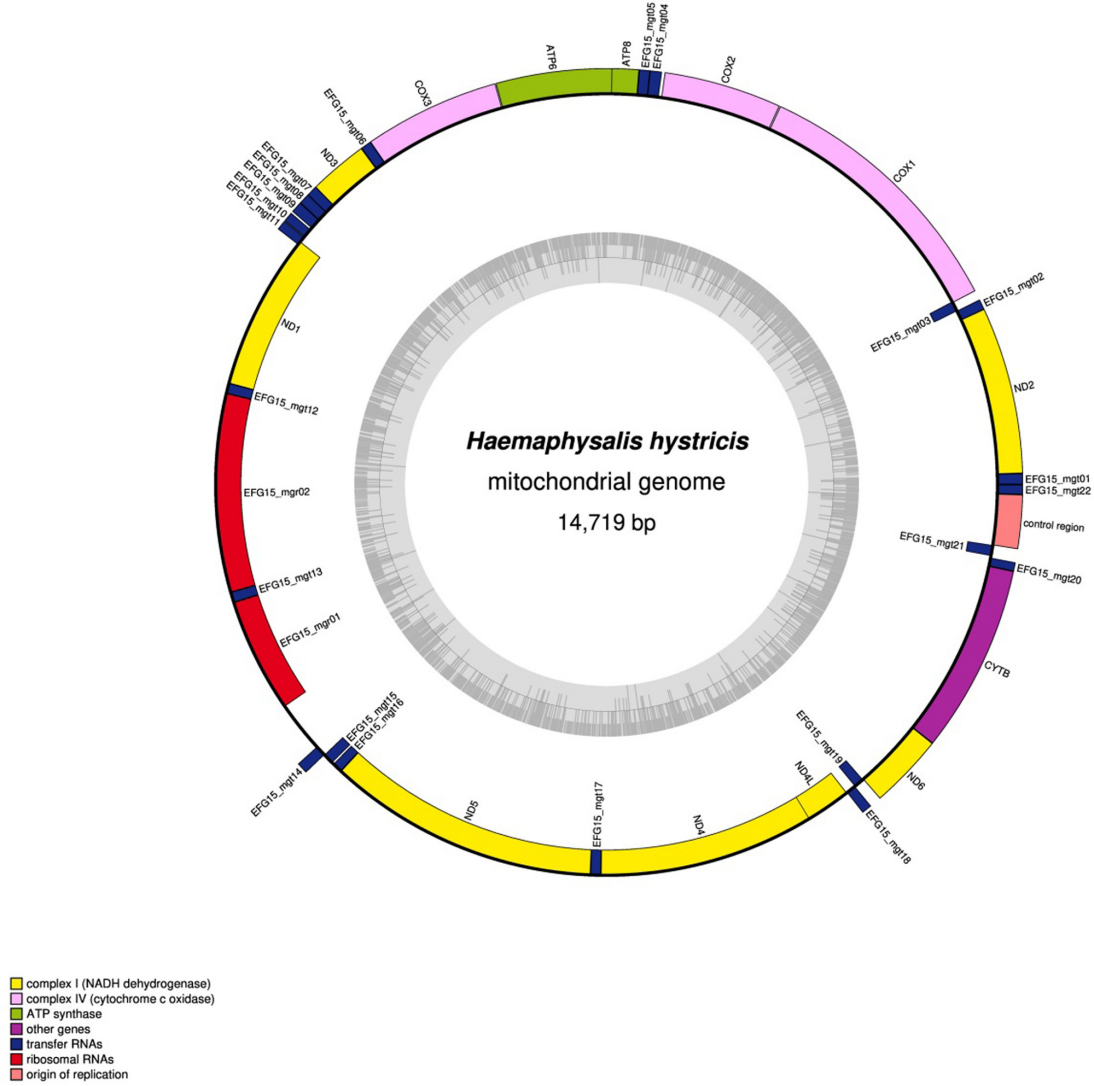
Complete mt genome of *H. hystricis*.

The complete mt genome of *H. hystricis* was 14,719 bp in size (Figure 1), and the mt genome sequence has been deposited in GenBank under the accession number PP396414. The mt genome of *H. hystricis* is a typical double stranded circular DNA molecule, which totally contains 37 genes including 13 PCGs, 22 tRNAs, 2 rRNAs, and 2 non-coding regions (Figure 1; Table 2). The gene order on the mt genome is identical to that of the ticks from Haemaphysalis family, but significantly different from that of soft ticks. All genes are transcribed in the same direction. The nucleotide composition of the entire mt genome of *H. hystricis* is *A* = 5,649 (38.4%), *T* = 5,719 (38.9%), *G* = 1,450 (9.9%), and *C* = 1,901 (12.9%), and its *A* + *T* content was 77.3%, *G* + *C* content was 22.8%, showing obvious base AT preference, in accordance with mt genomes of metazoan animals sequenced to date.

**Table 2:** Organization of *H. hystricis* mitochondrial genome

Gene	Position	Length (bp)	Start codons	Stop codons	Anti-codons
*tRNA*-*Met*	1–62	62			CAT
*nad2*	63–1,019	957	ATT	TAA	
*tRNA*-*Trp*	1,018–1,078	61			TCA
*tRNA*-*Tyr*	1,077–1,140	64			GTA
*cox*1	1,133–2,671	1,539	ATT	TAA	
*cox*2	2,676–3,350	675	ATG	TAA	
*tRNA*-*Lys*	3,367–3,434	68			CTT
*tRNA*-*Asp*	3,434–3,498	65			GTC
*atp8*	3,499–3,654	156	ATT	TAA	
*atp6*	3,647–4,314	668	ATG	TAA	
*cox3*	4,319–5,098	780	ATG	TAA	
*tRNA*-*Gly*	5,098–5,159	62			TCC
*nad3*	5,160–5,498	339	ATT	TAG	
*tRNA*-*Ala*	5,497–5,549	53			TGC
*tRNA-Arg*	5,558–5,621	64			TCG
*tRNA-Asn*	5,622–5,687	66			GTT
*tRNA-Ser1*	5,698–5,755	58			TCT
*tRNA-Glu*	5,762–5,815	54			TTC
*nad1*	5,793–6,749	957	ATT	TAA	
*tRNA-Leu1*	6,750–6,811	62			TAA
*rrnL*	6,812–8,017	1,206			
*tRNA-Val*	8,018–8,080	63			TAC
*rrnS*	8,082–8,775	694			
NCRS	8,776–9,083	308			
*tRNA-Ile*	9,084–9,150	67			GAT
*tRNA-Gln*	9,151–9,227	77			TTG
*tRNA-Phe*	9,228–9,288	61			GAA
*nad5*	9,291–10,942	1,652	ATT	TA	
*tRNA-His*	10,943–11,005	63			GTG
*nad4*	11,006–12,320	1,315	ATG	T	
*nad4L*	12,314–12,589	276	ATG	TAA	
*tRNA-Thr*	12,592–12,651	60			TGT
*tRNA-Pro*	12,652–12,714	63			TGG
*nad6*	12,717–13,146	430	ATC	T	
*cytb*	13,147–14,226	1,080	ATG	TAA	
*tRNA-Ser2*	14,227–14,277	51			TGA
*tRNA-Leu2*	14,289–14,353	65			TAG
NCRL	14,354–14,662	309			
*tRNA-Cys*	14,663–14,718	56			GCA

### 3.2 PCGs and codon usage

The mt genome of *H. hystricis* totally contains 13 PCGs including *nad2*, *cox1*, *cox2*, *atp8*, *atp6*, *cox3*, *nad3*, *nad1*, *nad5*, *nad4*, *nad4*L, *nad6*, and *cytb*, of which the longest sequence is *nad5* gene of 1,652 bp, and the shortest sequence is *atp*8 gene that has 155 bp in size. Moreover, in this mt genome, all PCGs used ATN as their initiation codon. For example, the *nad2*, *cox1*, *atp8*, *nad3*, *nad1*, and *nad5* genes start with ATT, the *cox2*, *atp6*, *cox3*, *nad4*, *nad4*L, and *cyt*b genes start with ATG, and the *nad6* use ATC, respectively (Table 2). In the same way, 10 of 13 PCGs, namely *nad2*, *cox1*, *cox2*, *atp8*, *atp6*, *cox3*, *nad3*, *nad1*, *nad4*L, and *cytb*, have complete termination codon (TAN), of which the *cox1*, *atp8*, *atp6*, *nad1*, *nad5*, *nad4*L, *nad6*, and *cytb* use TAA as termination codon, the *nad3* uses TAG as termination codon. Three of 13 PCGs including *nad5*, *nad4*, and *nad5* have incomplete termination codon (TA/T), of which *nad4* and *nad6* genes use T as termination codon, and the *nad5* gene uses TA as termination codon. Furthermore, all PCG sequences display base AT preference, which means that their AT content is significantly higher than the GC content.

### 3.3 Intergenic spacers and overlapping sequences

There are 13 gene gaps in the mt genome of *H. hystricis*, which varies from 1 to 16 bp. The longest gene gap is located between *cox*2 and tRNA-Lys. Similarly, eight gene overlap regions with different lengths are found in this mt genome, which varies from 1 to 23 bp. The longest gene overlap region is located s between *tRNA*-*Glu* and *nad1*. The length of all gene gaps is 386 bp accounting for 2.62%.

### 3.4 Transfer RNA genes (tRNA)

**Figure 2:**
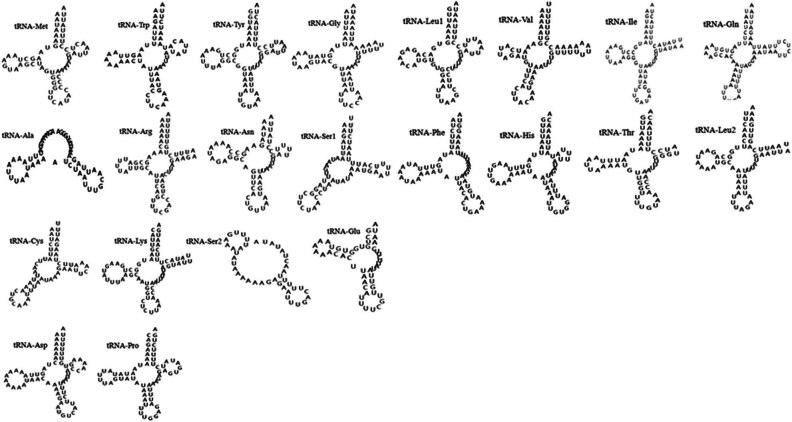
Putative secondary structures of the 22 tRNA genes of *H. hystricis*.

The mt genome of *H. hystricis* totally contains 22 tRNA genes that range from 51 (*tRNA*-*Ser2*) to 77 bp (*tRNA*-*Gln*) in size. These tRNAs gene orders were identified in other ticks from *Haemaphysalis* family mt genome. All 22 tRNA genes could form typical clover structures expect for *tRNA*-*Ala*, *tRNA*-*Ser1*, *tRNA*-*Ser2*, and *tRNA*-*Glu*. Their predicted secondary structures are shown in Figure 2, which are like that of other hard ticks. According to Figure 2, we found that the majority of tRNAs display base mismatch phenomenon such as U-U and G-U based on base complementary pairing principle. However, all tRNAs lack components of the secondary structure. For instance, *tRNA*-*Gly*, *tRNA*-*Arg*, *tRNA*-*Ile*, *tRNA*-*Leu2*, and *tRNA*-*Lys* lack a TψC loop, the *tRNA*-*Met*, *tRNA*-*Trp*, *tRNA*-*Asn*, *tRNA*-*Leu1*, *tRNA*-*His*, *tRNA*-*Asp*, and *tRNA*-*Pro* lack a TψC arm, the *tRNA*-*Ala* and *tRNA*-*Ser2* lack an amino acid acceptor arm, and all tRNAs of *H. hystricis* lack a variable loop.

### 3.5 Ribosomal RNA genes

The mt genome of *H. hystricis* contains two rRNAs with different sizes, namely *rrnL* and *rrnS*. The *rrnL* gene of *H. hystricis* is located between *tRNA*-*Leu1* and *tRNA*-*Val*, and *rrnS* gene is located between *tRNA*-*Val* and *tRNA*-*Ile*. The *rrnL* gene sequence of 1,206 bp is longer than the *rrnS* gene (694 bp) in size. The *A* + *T* contents of the *rrnL* and *rrnS* genes are 83.2 and 79.7%, respectively, which is similar to some of the other hard ticks.

### 3.6 *A* + *T*-rich region

The mt genome of *H. hystricis* contains two non-coding regions (*A* + *T*-rich region), namely the long non-coding region and the short non-coding region. The long non-coding region was named NCRL, and the short non-coding region was named NCRS in this study. For *H. hystricis*, the NCRL is located between the *tRNA*-*Leu2* and *tRNA*-*Cys*, and the NCRS is located between the *rrnS* and tRNA-Ile. The lengths of NCRL and NCRS are 309 and 308 bp, respectively. However, the *A* + *T* content of the NCRL and NCRS are 65.0 and 68.1%, respectively, which means that their sequences show base AT preference.

### 3.7 Phylogenetic analyses

**Figure 3:**
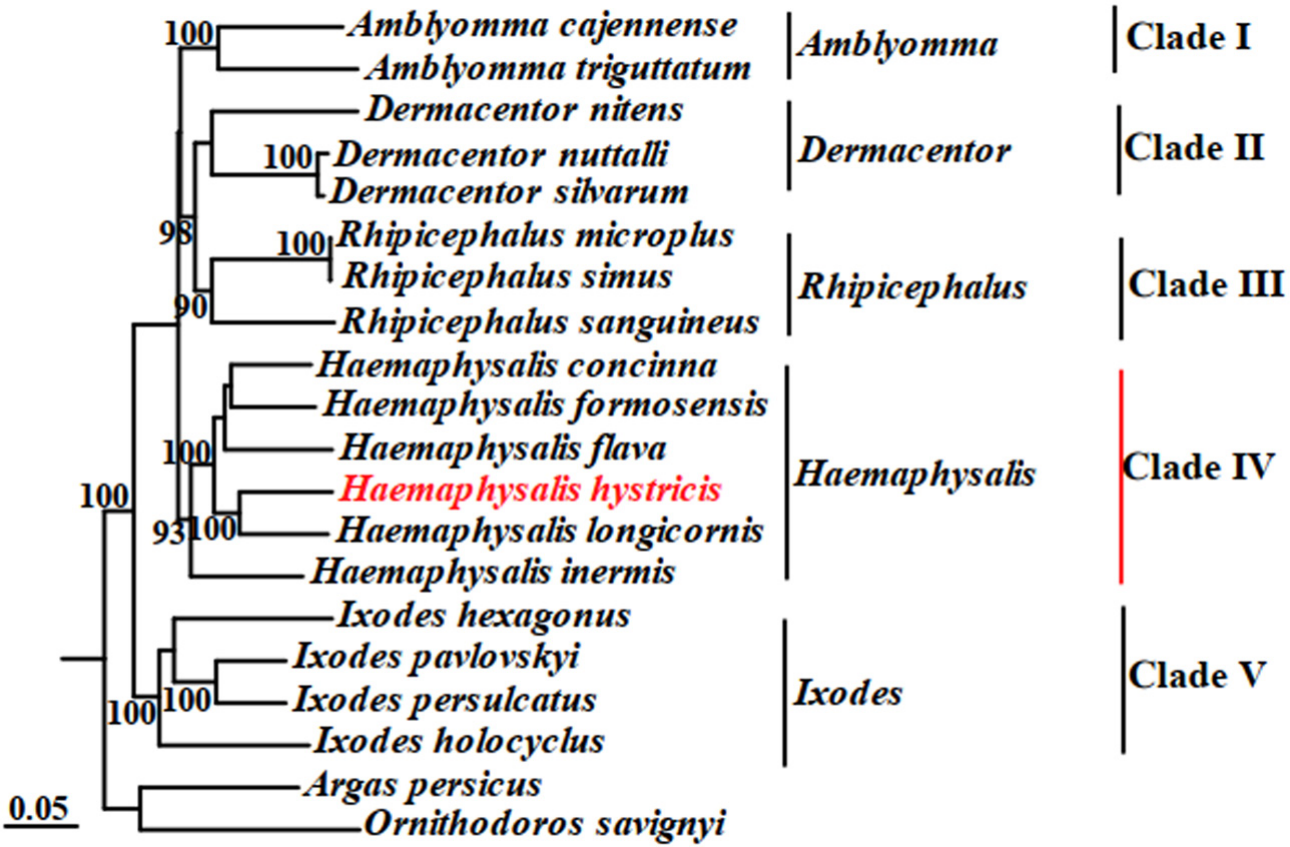
Phylogenetic tree (ML) of *H. hystricis* by concatenated sequences of 13 PCGs and 2 rRNAs.

**Figure 4:**
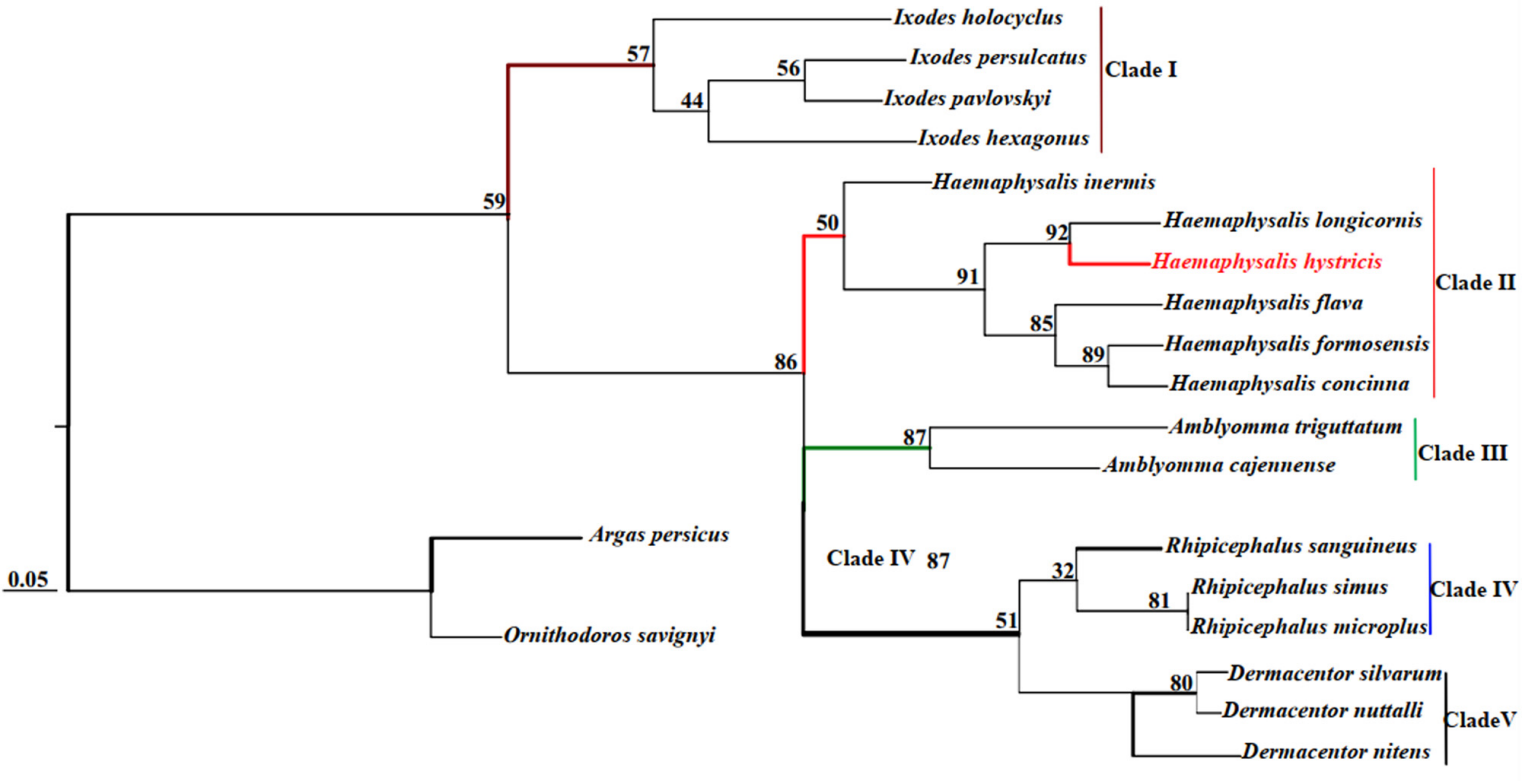
Phylogenetic tree (BI) of *H. hystricis* by concatenated sequences of 13 PCGs and 2 rRNAs.

Phylogenetic analyses of *H. hystricis* with selected 18 species of ticks from five genera were performed by ML and BI based on concatenated sequences of 13 PCGs and 2 rRNAs. The phylogenetic trees were reconstructed by computer programs Clustal X, PhyML 3.0, and FigTree v1.3.1, as shown in Figures 3 and 4. According to Figures 3 and 4, we observed that the phylogenetic trees were both divided into five major clades (Clade I–V). Within the two trees, *I. persulcatus*, *I. hexagonus*, *I. holocyclus*, and *I. pavlovskyi* together clustered into a branch, and the node values were 90 and 57 in the ML and BI trees, respectively; *D. nuttalli*, *D. silvarum*, and *D. nitens* also formed a branch; *A. cajennense* and *A. triguttatum* together formed a branch; *R. australis*, *R. simus*, *R. sanguineus*, and *R. microplus* together clustered into a branch; *H. hystricis*, *H. longicornis*, *H. formosensis*, *H. concinna*, *H. inermis*, and *H. flava* together formed a branch, but this branch was divided into three small branches again, *H. concinna*, *H. formosensis*, and *H. flava* together formed the first small branch, *H. longicornis* and *H. hystricis* together formed the second small branch, *H. inermis* individually formed the third small branch, respectively. Based on the phylogenetic analyses results, we found that *H. hystricis* was more closely related to *H. longicornis* than the other selected species of ticks, which is consistent with that of the study on ticks classification [40].

## 4 Discussion

In the recent years, as the study on the classification and identification of parasites has developed toward the molecular level, many molecular biology techniques were applied widely to parasitology research, especially PCR. Compared to traditional morphological methods, not only the molecular biology technology is completely unaffected by individual differences, sample integrity, appraiser experience, developmental stages of the insect, and its environment, but also the classification and identification results obtained have a high degree of accuracy [41]. Currently, molecular biology technology has become an important auxiliary means for classification and identification of parasites [42–44]. The most crucial step in using molecular biotechnology to identify and classify parasites is how to find and select appropriate genetic markers. According to the massive research literature, mt genome of metazoan animals and some of its gene sequence all have characteristics such as rapid genetic evolution, lack of gene recombination, and small intra-species differences but large inter-species differences [28,31]. Due to the information provided by the entire mt genome is far more than that of fragmented gene sequences, so some researchers think that the mt genome is more valuable as genetic marker for studying species identification, genetic structure, gene variation, haplotype polymorphism, and phylogenetic relationship of parasites [24,45]. Thus, we sequenced the mt genome of *H. hystricis* by PCR, and analyzed its sequence characteristics, gene composition, arrangement order, and codons usage; meanwhile, we explored phylogenetic relationship between *H. hystricis* and other ticks by 13 PCG sequences.

Based on the sequencing, annotation, gene composition, and arrangement order results, the entire mt genome of *H. hystricis* contains 37 genes (13 PCGs, 22 tRNAs, 2 rRNAs), which is in accordance with other metazoan animals’ mt genome reported previously [46–48]. Compared with other ticks from *Haemaphysalis* family, we found that the mt genome of *H. hystricis* is far longer than that of *H. longicornis*, *H. formosensis, H. concinna*, and *H. flava*, but is significantly shorter than that of *H. inermis*, which may be closely related to the interval region, overlapping sequence, and D-loop region contained in their mt genome. Generally, most of the scholars believed that the number and length of D-loop region contained in the mt genome directly affect the length of the mt genome [49,50]. However, some scholars opined that the D-loop region sequence in mt genome lose bases, which affects the length of the mt genome [51,52], but Zhang and Hewitt thought that the number of concatenated duplicate copies in D-loop region directly affect the length of the mt genome [53]. However, the mt genome of *H. hystricis* and its 13 PCG sequences both show base AT preference clearly, which is consistent with that of the results of the sequence characteristics of mt genome of metazoan animals [54,55]. Some researchers pointed out that the mt genome of metazoan animals has the characteristics of multiple copy numbers and fast evolution rate [28,31], which may be related to its base AT preference. To the best of our knowledge, a nucleotide sequence has high AT content meaning that it has a low melting point temperature causing DNA double stranded to dissociate into single strand, which greatly increases the probability of base mutation and improves the speed of evolution.

According to the results of the codons usage, we found that all PCGs used a standard ATN start codon in mt genome of *H. hystricis.* Six PCGs (*nad2*, *cox1*, *atp8*, *nad3*, *nad5*, and *nad6*) started with ATT (Ile), five PCGs including *cox2*, *atp6*, *cox3*, *nad4*, and *nad4L* started with ATG (Met), and *nad1* started with ATA (Met), which is consistent with other ticks [28,56,57]. All initiation codons of this mt gene also appear in other mt gene of other arthropods including most Acari species, which suggests that the pattern of initiation codon usage is relatively conservative in arthropod mitochondrial genomes [58]. However, those unusual start codons such as TTG and GTG that are predicted in *D. pteronyssinus* and *D. farinae* [57] are not found in *H. hystricis*, suggesting that the mt genome of *H. hystricis* is no alternative start codons. A total of eight PCGs applied the complete termination codon (TAA and TAG), while four PCGs (*nad2*, *cox3*, *nad3*, and *nad4*) ended at a single T residue, and the cytb ended at TA residue, which is also consistent with some ticks and arthropod [28,57]. The mitochondrial genome of metazoans with incomplete stop codon is common, and some studies believed that these incomplete stop codons are the results of gene polyadenylation after transcription [28,57,59]. In *H. hystricis*, the PCGs that use T as termination codon may end with a TGA codon; however, in this gene, as the GA nucleotide in the termination codon overlaps with the 5′end of downstream genes, they are annotated as ending with the T to minimize overlapping [57]. Usually, the termination codon of the *cyt*b gene in mt genome of metazoans is considered to be complete by cleaving the transcripts of the polycistron, and form the stop codon by mRNA polyadenylation [60].

According to the results of the tRNAs analysis, we found that most of tRNAs (except for *tRNA*-*Ala*, *tRNA*-*Ser1*, *tRNA*-*Ser2*, and *tRNA*-*Glu*) have typical secondary structures, but five tRNAs lack a TψC loop, and all tRNAs lack the variable loop. Generally, the tRNA losing the D-arm is common, especially for the *tRNA*-*Ser1*, *tRNA*-*Ser2*, *tRNA*-*Leu1*, and *tRNA*-*Leu2*. Some studies pointed out that the *trnS1* lost D-arm has been considered as a typical feature in mt genome of all chelicerate [61]. The tRNA-Ser1 in the mt genome of *H. hystricis* does not lose the D-arm, which agrees with the viewpoint that this structure in other tRNAs of arthropods is less common as proposed by Sun et al. [57]. Besides, all tRNAs of *H. hystricis* do not lose the D-arm, which is contrary to those study results that tRNAs lacking D-arm were found in mt genome of the sea spiders, the scorpion, Ixodida, Mesostigmata, and Acariformes. Furthermore, we found that the majority of tRNAs in *H. hystricis* exhibits base mismatch phenomenon (U-U and G-U), which is consistent with the results reported previously [31,56]. Some researchers opined that the base mismatch may be closely related to the rare base in tRNAs [62].

According to the results of the rRNAs analysis, we found that the *rrnL* is longer than *rrnS* in size, and both are located in close proximity to each other on the N-strand and next to the largest non-coding regions, which is consistent with many mt genome of arthropods including *H. longicornis*, *H. formosensis*, *H. concinna*, *H. inermis*, and *H. flava* reported previously [53], but is significantly different from the mt genome of *D. pteronyssinus*, *D. farinae*, and *P. persimilis*. The *rrnL* and *rrnS* genes in the mt genome of *D. pteronyssinus*, *D. farinae*, and *P. persimilis* are both located on the J- strand, very close to each other, and distant from the largest non-coding regions [63,64]. These results suggested that the transcription mechanisms of rRNA gene of metazoans may be different, and it requires further investigation. Besides, the sequences of *rrnS* and *rrnL* both show AT preferences.

According to the results of the non-coding regions analysis, we found that there are gene gaps, overlapping regions, and two non-coding regions in *H. hystricis* mitogenome. However, the number of gene gap is far more than that of overlapping region, which suggests that mt genome of *H. hystricis* is not very tight. Two non-coding regions, also known as D-loop region, in the mt genome of *H. hystricis* have different lengths, and both display AT preferences. Some researchers point out that the non-coding region of arthropods is rich in tandem AT repeat sequences, its *A* + *T* content ranges from 80 to 98% [65], and these AT repeat sequences directly affect the entire mitochondrial genome in size. Moreover, the non-coding region contains a large number of replication starting points, transcription starting points, and control sequences [66], which forms a secondary structure of the stem ring through hydrogen bonding, and there are highly conserved sequences related to the replication, transcription, and translation processes of the mitochondrial genomes such as TATA and GAAT on both sides of the stem ring structure.

According to the results of the phylogenetic analysis, we found that there is a close phylogenetic relationship between *H. hystricis* and *H. longicornis* among the selected ticks from *Haemaphysalis* family, followed by *H. flava*, *H. formosensis*, *H. concinna*, and *H. inermis*. Compared to other genus ticks, the phylogenetic relationship of *Haemaphysalis* and *Amblyomma* ticks is the closest, followed by *Dermacentor* and *Rhipicephalus* genera, and the Ixodes genus is the latest. It suggests that the mt genome of *H. hystricis* is an effective tool for studying its molecular epidemiology, population genetics, and systematics. Sequencing mt genome of *H. hystricis*, not only enriches the genome database, but also has implications for the diagnosis, prevention, and control of ticks and tick-borne diseases in animals and humans.

## 5 Conclusion

In the present study, we determined the complete mtDNA data of *H. hystricis* and compared with that of other ticks from *Haemaphysalis* and other genera. According to the results, we found that the mt gene arrangement for *H. hystricis* is the same as that of the selected ticks from *Haemaphysalis* family, and its sequence shows AT preference clearly. Most of the tRNA genes of *H. hystricis* are able to form typical clover structures expect for *tRNA*-*Ala*, *tRNA*-*Ser1*, *tRNA*-*Ser2*, and *tRNA*-*Glu*, and there are base mismatch phenomenon in the structures of tRNAs. The mt genome of *H. hystricis* has two rRNAs and non-coding regions of distinct AT preference. Phylogenetic analyses also indicate that *H. hystricis* is more closely related to *H. longicornis* than to other ticks. However, the mt genome has implications for further studying the molecular identification, population genetics, systematics of ticks, as well as the diagnosis, prevention, and control of tick-borne diseases in animals and humans.
